# Associations between Obesity and Spinal Diseases: A Medical Expenditure Panel Study Analysis

**DOI:** 10.3390/ijerph14020183

**Published:** 2017-02-13

**Authors:** Binwu Sheng, Chaoling Feng, Donglan Zhang, Hugh Spitler, Lu Shi

**Affiliations:** 1 First Affiliated Hospital of Xi’an Jiaotong University, Xi’an 710061, China; 2 Samuel Curtis Johnson Graduate School of Management, Cornell University, Ithaca, NY 14853, USA; chaoling.feng@gmail.com; 3 Department of Health Policy and Management, University of Georgia, Athens, GA 30609, USA; dzhang@uga.edu; 4 Department of Public Health Sciences, Clemson University, Clemson, SC 29631, USA; HSPITLE@clemson.edu (H.S.); lus@clemson.edu (L.S.)

**Keywords:** obesity, low back pain, disc degeneration, spondylosis, cervical diseases, spinal disease

## Abstract

*Background:* The link between body weight status and spinal diseases has been suggested by a number of cross-sectional and cohort studies with a limited range of patient populations. No population-representative samples have been used to examine the link between obesity and spinal diseases. The present study is based on a nationally representative sample drawn from the Medical Expenditure Panel Survey. *Methods:* Using the cross-sectional sample of the 2014 Medical Expenditure Panel Study, we built four weighted logistic regression analyses of the associations between body weight status and the following four spinal diseases: low back pain, spondylosis, other cervical disorders and intervertebral disc disorder (IDD). Each respondent’s body weight status was used as the key independent variable with three categories: normal/underweight, overweight, and obese. We controlled for marital status, gender, age, smoking status, household income, health insurance coverage, educational attainment and the use of health services for other major categories of diseases. *Results:* A total sample of 23,048 respondents was used in our analysis. Overweight and obese respondents, as compared to normal/underweight respondents, were more likely to develop lower back problems (Overweight: logged odds = 0.218, *p* < 0.01; Obese: logged odds = 0.395, *p* < 0.001) and IDD (Overweight: logged odds = 0.441, *p* < 0.05; Obese: logged odds = 0.528, *p* < 0.001). The associations between bodyweight status and spondylitis were statistically insignificant (Overweight: logged odds = 0.281, *p* = 0.442; Obese: logged odds = 0.680, *p* = 0.104). The associations between body weight status and other cervical disorders (Overweight: logged odds = −0.116, *p* = 0.304; Obese: logged odds = −0.160, *p* = 0.865) were statistically insignificant. *Conclusions:* As the first study using a national sample to study bodyweight and spinal diseases, our paper supports the hypothesis that obesity adds to the burden of low back pain and IDD. Longitudinal and interventional studies are needed to understand the specific mechanisms behind these positive associations.

## 1. Introduction

Obesity is a rising public health concern in the U.S [1,2]. Ogden et al. [3] reported that the prevalence of obesity in the U.S. was 36.5% among adults and 17% among children and youth during 2011–2014. Obesity is associated with many prevalent conditions including prostate disease [4], cardiovascular diseases [5], diabetes [6], and osteoarthritis [7,8]. A number of studies have found a consistent association between obesity and low back pain (LBP) [9,10,11,12,13]. Koyangi, et al. [9] conducted a cross-sectional study of data collected from nine countries as part of the Collaborative Research on Ageing in Europe (COURAGE) study and found significantly higher odds for LBP among those with higher levels of body mass index (BMI). Similar associations between high levels of body mass index (BMI) and LBP were found by Heuch et al. [14] while Smuck et al. [15] found the risk of LBP increases as BMI increases. Among those suffering from chronic pain disorders, pain arising from various structures of the spine accounts for a majority of those affected and the lifetime prevalence of spinal pain has been reported as ranging from 54% to 80% [16]. LBP has tended to be the leading cause of disability worldwide [17]. In the U.S., LBP and costs related to treatment of LBP are escalating and the prevalence of LBP continues to increase [18]. Ferreira et al. [13] and Shiri et al. [11] found a dose-response relationship between obesity and low back pain—the more obese the individual the higher the odds for LBP and the greater the intensity of back pain.

Studies conducted by Heuch et al. [14], Smuck et al. [15] and Wertli et al. [19] found that high values of BMI consistently predispose individuals to chronic LBP. For instance, in Heuch et al.’s study [14], the odds ratio for BMI 30 or more vs. BMI less than 25 was 1.34 (95% confidence interval (CI), 1.08–1.67) for men and 1.22 (95% CI, 1.03–1.46) for women after adjusting for confounding factors. Knutsson et al. [20] found a linear positive relationship between BMI and lumbar spinal stenosis. Several studies suggest that high BMI and obesity may be linked to lumbar disc degeneration [17,21,22,23,24,25,26]. Takatalo et al. [25] note that BMI is commonly used as a standardized measure to assess overweight and obesity, although BMI does not indicate specifically the distribution of body fat and muscle mass. In the majority of the studies cited in this review, BMI was calculated as weight in kilograms divided by height in meters squared using the standard World Health Organization (WHO) definition [9,11,25]. Studies using BMI as an index of obesity specify cutoffs indicating levels of obesity, with overweight typically ranging from 25–29.9 kg/m^2^ and obesity as ≥30 kg/m^2^. Obesity, particularly the distribution of adiposity in the trunk of the body, is strongly linked to biomechanical changes that damage the spine and contribute to a range of spinal diseases including intervertebral disc degeneration, spinal stensosis, reduce disc height, herniation of the disc, hypertrophy of the spinal ligaments, osteoarthritis, and increased compression forces on disc surfaces [20,27,28,29]. To more fully explore in impact of obesity on the entire spine, Teraguchi et al. [24] investigated the prevalence and distribution of intervertebral disc degeneration over the entire spine and found the age and obesity were associated with the presence of disc degeneration in all areas of the spine, indicating that obesity places stress across multiple regions of back. BMI and obesity have also been identified as a risk factor for adjacent segment diseases and post-operative complications among patients undergoing lumbar fusion for degenerative spine diseases [30,31,32,33,34,35,36,37,38,39,40]. Weight control before and after the surgery was observed to reduce the incidence of adjacent segment disease and improve the fusion surgery outcome [41,42].

Thus far, research on the link between obesity and spine diseases has relied on exploratory studies used small, unrepresentative patient samples, and narrowly focused on lumber disc degeneration [21,22,43,44,45]. In this study, we analyzed 2014 Medical Expenditure Panel Study (MEPS) data, a nationally representative dataset based on the U.S. population, to explore the association between BMI and the presence of a broad range of spine disc degeneration conditions, including intervertebral disc disorder, spondylosis, other cercal and lower back degeneration.

## 2. Materials and Methods

We analyzed the cross-sectional sample of the 2014 Medical Expenditure Panel Study (MEPS) [46] to explore the association between adult’s bodyweight status and spinal diseases. We chose the data source of MEPS since its cross-sectional sample provides a representative sample for the U.S. population, and contains information about the International Classification of Diseases (ICD) codes indicated in the description of the respondents’ patterns of health care utilization. The dataset was publicly available with survey weights calculated to facilitate the analysis.

With survey weights provided by MEPS, implemented through STATA’s svy command [47] (Stata Corp., College Station, TX, USA), we built four weighted logistic regression analyses of the following four spinal diseases: low back pain (ICD 9: 724), spondylosis (ICD 9: 721), other cervical disorders (ICD 9: 723) and intervertebral disc disorder (ICD 9: 722). We add a fifth weighted logistic regression model with the binary dependent variable built as “any spinal disease”: a patient who had any of the four conditions was coded as 1 in this variable and a patient who had none of the four conditions was coded as 0. In each of the five weighted logistic regressions, each respondent’s body weight status was used as the key independent variable with three BMI-based categories: normal or under (BMI < 25), overweight (25 ≤ BMI < 30), and obese (BMI > 30). This is consistent with the categorization in previous studies such as Heuch et al. [14].

Using a weighted logistic regression model we also controlled for marital status, gender, age, smoking status, household income, health insurance coverage, educational attainment and the use of health services for other major categories of diseases (diabetes, mental disease, skin disease, cancer, asthma and pneumonia).

## 3. Results

A total of 23,048 cases were used in our analyses due to the missing values in the 2014 MEPS cross-sectional dataset. The descriptive statistics of the independent variables and dependent variables used in our logistic regression analyses were charted by Table 1 and Table 2. As Table 2 shows, of the four conditions we studied, low back pain was the most common problem (7.4%), following by intervertebral disc disorder (1.7%), other cervical disorder (1.3%) and spondylosis (0.2%). In total, 9.7% of the sample has at least one of the four spinal diseases.

The logistic regression analyses (Table 3) showed that overweight and obese respondents, as compared to normal or underweight respondents, were more likely to develop lower back problems (Overweight: Odds ratio = 1.244, *p* < 0.01; Obese: OR = 1.484, *p* < 0.001) and intervertebral disc disorders (Overweight: OR = 1.554, *p* < 0.05; Obese: OR = 1.696, *p* < 0.001). The association between bodyweight and spondylosis, though also positive, was statistically insignificant (Overweight: OR = 1.324, *p* = 0.442; Obese: OR = 1.974, *p* = 0.104). The association between obesity and other cervical disorders (Overweight: OR = 0.890, *p* = 0.304; Obese: OR = 0.852, *p* = 0.865) is also insignificant.

When we used the recoded measure of “having any spinal disease” as the dependent variable of a weighted logistic regression, both overweight (OR = 1.257, *p* < 0.001) and obese (OR = 1.456, *p* < 0.001) had a statistically significant positive association with this outcome variable.

## 4. Discussion

Our finding about the association between obesity and spinal diseases was consistent with findings from prior studies [48,49,50,51,52]. In one prior study, a modest but positive association between obesity and low back pain (LBP), in particular chronic LBP, was identified in a cross-sectional survey data study of 29,424 twin subjects [53]. Among patients diagnosed with spinal diseases, higher BMI was associated with increased disability, more severe pain symptoms, and comorbid conditions [54]. Our study did not find significant correlation between obesity and cervical diseases, which was also consistent with findings of prior studies [34,55,56,57,58]. This could possibly be explained by the fact that cervical segment need not bear as much bodyweight as the lower segments such lumbar segments [59,60], while it is also possible that the small sample size of cervical diseases (as seen even in our large-sample study) does not provide enough power to detect any significant association in most research samples. Using STATA’s module of powerlog [61], our power analysis shows that a sample size of 213,081 is needed to detect the significant pattern between spondylosis and overweight status. However, in our study the highly significant associations between higher bodyweight status and “having any spinal disease” suggests that the significant patterns between those specific conditions and bodyweight status are unlikely to be a mere statistical anomaly.

The possible effect of obesity on degenerative disc diseases (DDD), as observed in our study, could be exerted through several structural mechanisms. Obesity could result in serious postural changes that affect loading on joints, and thus result in long-term adverse effects on bones and joints [62,63]. Increased body mass index increases lumbosacral angles, which results in biomechanical changes in the lumbosacral spine resulting in greater flexion of the sacroiliac joints, greater facet degeneration, higher torque on the lumbar discs and joints, and increasing sheer forces that may overload the joints [28,29]. These biomechanical changes may produce higher compressive forces contributing to LBP. Obesity could also induce other mechanical-structural alterations, including joint misalignment [64,65], and decreased ambulation and conditioning [66,67].

The metabolic mechanism could also be affected via the linkage between obesity and pain disorders. Whether obesity is an independent risk factor for the development of neuropathic pain disorders is not fully understood, but it is known that overweight individuals have an increased risk of various metabolic disorders, which could lead to increased risks of neuropathic disorders associated with conditions such as diabetes [68]. In other words, obesity and its associated metabolic disorders may increase the risk of peripheral neuropathic disorders [69]. In one study, after controlling for other major risk factors including duration of Type 1 diabetes and glycosylated hemoglobin values, diabetes patients with higher BMI was also found to have higher cumulative incidence of neuropathic conditions [70,71].

In addition, a possible link between obesity and disc degeneration of the spine could also be found among obese patients with the chronic inflammatory conditions. Obesity has been found to be associated with a chronic low-grade inflammatory response characterized by abnormal cytokine production, increased acute phase reactants, as well as activation of inflammatory signaling pathways in the adipose tissue [72], and it has been observed that expansion of adipose tissue during weight gain is linked with inflammatory macrophages through chemokines. Recent reports indicate that adipose tissue functions as an endocrine organ in which adipocytes and recruited macrophages produce cytokines such as tumor necrosis factor (TNF), interleukin 6 (IL-6) and adipokines such as adiponectin, leptin, and resistin, which are thought to be associated with obesity, insulin resistance, and other inflammatory disorders [73]. Leptin and resistin are pro-inflammatory, and have a strong effect on increasing IL-2 secretion and proliferation and memory T cells to produce interferon γ production, which could further aggravate lower disc degeneration [74,75]. Obesity has also been suggested to be correlated with cartilage inflammation [76]. From a genetic perspective, patients with DDD have low-grade systemic inflammation [77], while fat mass and obesity-associated gene (FTO) is an IDD predisposition gene and may lead to a positive correlation between obesity and DDD. In a study of a Chinese Han population, it has been suggested that the single nucleotide polymorphisms rs11076008 of FTO may have played an important role in the development of DDD and IDD [78,79]. Thus, obesity has been linked to both biomechanical and metabolic changes that contribute to LBP.

Our study has several limitations. Although the size and breadth of our sample provides a reasonable estimation of the impact of obesity on a selected range of spinal diseases, the precision and accuracy of our analysis depends on the accuracy of the ICD-9-CM coding. Recent studies in human and animal models have suggested that validating ICD coding with imaging data could make a strong study [69,73,80,81], as these studies address the issue of possible coding errors. Second, the obesity-DDD association from this study should be interpreted with caution, since it does not reject the “reverse causality” hypothesis (the spinal conditions could increase the risk for obesity as juvenile disc degeneration was strongly associated with diminished physical functioning [6] and diminished physical functioning could mean less energy expenditure). Moreover, with available evidence suggesting a likely relationship between increased BMI and a variety of pain conditions, the question remains as to whether the link we identified here was specific to spinal conditions [82]. It is possible that individuals who experienced ongoing pain may reduce their activity levels and therefore experience weight gain and eventual deconditioning, which might lead to a vicious circle of further increasing pain. Data with longitudinal follow-up about the physical activity information will be needed to examine this possible pathway between spinal diseases and obesity.

The use of MEPS data limits the generalizability of our results to the civilian non-institutionalized population in the U.S. Excluded from this database are those residing in nursing homes or long term care facilities, who may represent a high percentage of those suffering from chronic spinal disorders? The results of this study may underestimate the costs and impact of obesity on spinal diseases resulting in LBP.

## 5. Conclusions

This is the first study using population-representative national data to show that the intervertebral disc disorder and chronic LBP are linked with obese and overweight bodyweight status. Although we are unable to tell the exact causal mechanism behind these associational patterns given the cross-sectional nature of our data, our finding that obesity predicts spinal diseases in the lower back but not in the cervical region provides more support for the mechanical-structural hypothesis than for metabolic or behavioral hypothesis. From a policy and management viewpoint, if the causal link between spinal disease and obesity is further substantiated by longitudinal and interventional studies, the health care expenditure associated with obesity might be even higher than the current estimates [83,84,85,86] and thus the cost-benefit ratio of obesity interventions might be more favorable that what has been estimated so far [87,88,89,90,91]. In other words, further research is needed where researchers employ longitudinal analyses or interventional studies examining whether weight loss leads to improvement of spinal conditions. This would more precisely determine the links between levels of obesity and specific spinal conditions, as well as a fuller understanding of the benefits from obesity interventions.

## Figures and Tables

**Table 1:** Descriptive statistics about independent variables in the analysis sample (N = 23,048).

Variable	Percentage	Mean (Standard Deviation)
Age		46.0 (17.4)
Female	52.2%	
Living a metropolitan statistical area	88.2%	
Married	50.9%	
Not smoking	82.2%	
Diabetes	11.0%	
Mental disease	17.0%	
Skin problem	8.1%	
Pneumonia	1.3%	
Asthma	17.4%	
Cancer	5.7%	
***Insurance status***		
Private insurance	56.7%	
Public insurance	21.3%	
No insurance	22.0%	
***Region***		
Northeast	16.1%	
Midwest	18.5%	
South	38.3%	
West	27.1%	
***Race/ethnicity***		
Latino	27.7%	
Non-Latino White	42.7%	
Non-Latino Black	19.9%	
Asian	9.7%	
***Educational attainment***		
Below high school	21.1%	
High school diploma only	30.1%	
Some college	26.1%	
College or above	22.8%	
***Household income***		
≤Federal poverty line (FPL)	24.5%	
125%–200% of FPL	16.9%	
200%–400% of FPL	29.5%	
Above 400% FPL	29.0%	
***Weight status***		
Normal or underweight	33.8%	
Overweight	34.5%	
Obese	31.7%	

**Table 2:** Percentage of Spinal Diseases in the Analysis Sample (N = 23,048).

	Any Spinal Disease	Low Back Pain	Spondylosis	Other Cervical Disorder	Intervertebral Disc Disorder
Percentage	9.7%	7.4%	0.2%	1.3%	1.7%

**Table 3:** Weighted logistic regressions of bodyweight status and spinal diseases (N = 23,048).

Variables		Any Spinal Disease	Lower Back Pain			Spondylosis	Other Cervical Disorder	Intervertebral Disc Disorder
		Odds Ratio	Odds Ratio			Odds Ratio	Odds Ratio	Odds Ratio
Age		1.012 ***	1.012 ***			1.029 **	1.009 *	1.009 **
		(0.000)	(0.000)			(0.006)	(0.016)	(0.007)
Race (Latinos as reference)								
White	1.327 ***			1.169 *		5.709 *	1.613 **	2.020 ***
	(0.000)			(0.040)		(0.022)	(0.010)	(0.000)
Black	0.993			0.958		4.137	0.762	1.489 *
	(0.932)			(0.626)		(0.080)	(0.268)	(0.049)
Other	1.029			0.985		2.901	1.035	1.616
	(0.775)			(0.890)		(0.295)	(0.895)	(0.054)
Education (below school as reference)								
High school	1.181 *			1.151		0.376	1.331	1.366
	(0.023)			(0.084)		(0.061)	(0.170)	(0.069)
Some college	1.334 ***			1.311 **		1.155	1.502	1.449 *
	(0.000)			(0.001)		(0.755)	(0.055)	(0.039)
College & above	1.336 ***			1.328 **		0.459	1.422	1.522 *
	(0.000)			(0.002)		(0.168)	(0.122)	(0.031)
Insurance (private as reference)								
Public insurance	1.122			1.165 *	0.619		0.742	1.338 *
	(0.078)			(0.037)	(0.263)		(0.098)	(0.039)
No insurance	0.774 ***			0.798 **	0.153		0.744	0.803
	(0.001)			(0.008)	(0.070)		(0.141)	(0.227)
Not smoking	0.737 ***			0.783 ***	0.485 *		0.890	0.535 ***
	(0.000)			(0.000)	(0.038)		(0.465)	(0.000)
Not married	0.857 **			0.870 *	0.899		1.020	0.778 *
	(0.002)			(0.013)	(0.744)		(0.876)	(0.025)
Weight (normal/under as reference)								
Overweight	1.257 ***			1.244 **	1.324		0.890	1.554 **
	(0.000)			(0.001)	(0.504)		(0.426)	(0.001)
Obese	1.456 ***			1.484 ***	1.974		0.852	1.696 **
	(0.000)			(0.000)	(0.091)		(0.293)	(0.000)

Notes: Standard errors of logistic regression coefficients are in parentheses. *p*-values in parentheses: * *p* < 0.05, ** *p* < 0.01, *** *p* < 0.001. The models also control the four geographical regions and whether the respondent lived in a metropolitan statistical area, plus the respondent’s utilization of health care.
